# Dietary Drivers and Challenges of Australian Breast Cancer Survivors: A Qualitative Study

**DOI:** 10.1089/whr.2021.0133

**Published:** 2022-06-10

**Authors:** Daniel G. Coro, Amanda D. Hutchinson, Siobhan Banks, Alison M. Coates

**Affiliations:** ^1^ Behaviour-Brain-Body Research Centre, UniSA Justice & Society, University of South Australia, Adelaide, Australia.; ^2^ Alliance for Research in Exercise, Nutrition and Activity (ARENA), UniSA Allied Health & Human Performance, University of South Australia, Adelaide, Australia.

**Keywords:** barriers, cancer survivors, cognition, diet, nutrition, qualitative

## Abstract

**Purpose:**

Cancer survivors often make long-term dietary changes, and nutrition is important for survivorship outcomes. Many survivors experience persisting cognitive difficulties, which can impact health behaviors. This study aimed to identify perceived drivers of eating habit changes, and the barriers to making intentional dietary changes, among breast cancer survivors with persisting self-reported cancer-related cognitive impairment.

**Materials and Methods:**

A qualitative framework explored survivors' perceptions of dietary habit changes. Thirteen Australian breast cancer survivors (M.time since diagnosis: 23.6 months, standard deviation [SD] 15.3; M.time since completing primary treatment: 14.7 months, SD 15.3) completed semistructured interviews. Questions related to dietary changes since diagnosis and treatment. Major themes were identified from interview transcripts using thematic analysis.

**Results:**

While most individuals perceived their diet to be broadly similar to prediagnosis, several changes to diet and eating habits were identified, which were often meaningful to these survivors. Themes relating to survivors' eating habit changes included the following: (1) meal timing and frequency shifts, (2) more plant-based eating, and (3) less variety and more convenience. Changes in eating habits were attributed to the following: (1) persisting treatment-related changes, (2) help and support from others, (3) old treatment habits, (4) preventative health and self-care, and (5) changes to work schedule. Barriers to making intentional dietary changes included the following: (1) too much time and effort, (2) food cravings and enjoyment, and (3) lacking dietary ideas and resources.

**Conclusions:**

Many survivors reported long-term changes in dietary habits, some of which align with current recommendations. Causes of dietary habit changes, and barriers to engaging in healthier dietary habits, involved multiple biopsychosocial elements. Additional resources or strategies that assist navigating survivorship challenges and their effects on dietary habits are needed. Future studies should explore whether post-treatment nutritional review with a qualified dietary health professional is helpful for survivors who experience long-term cancer-related cognitive impairment.

## Introduction

Cancer is the leading disease group contributing to disability-adjusted life years (a measure of years of potential life and productivity lost from an illness) in Australia, placing a significant burden on society.^1^ Dietary factors are the second-most important modifiable factor in the prevention of cancer incidence and mortality.^2^ Cancer survivors are advised to follow the dietary recommendations for cancer prevention for the general population; this includes the following: (1) a diet rich in whole grains, vegetables, fruits, and beans, (2) limiting “fast foods” and foods high in fat or sugar, and (3) limiting red meat.^3^

During active treatment, survivors commonly make dietary changes, often due to treatment-related side effects such as fatigue, nausea, and taste perception changes.^4^ However, nutrition is an area of interest and importance to survivors of cancer,^5^ who often make intentional long-term dietary changes following treatment.^6^ The importance of supporting and promoting healthier lifestyle behaviors in cancer survivors has been emphasized in prior research.^7,8^

An important facet to improving survivors' lifestyle habits is to understand factors that influence dietary changes or prevent engaging in positive dietary habits, at the outset. Previous qualitative research has touched upon this topic. One study in 2001 explored the causes of dietary changes in mixed cancer populations.^9^ They most commonly reported increases in vegetable and fruit intake (for 67% and 45%, respectively) and a decrease in meat intake (for 58%). Reasons for dietary changes included improving or maintaining health.

Another study in 2017 looked at healthy lifestyle challenges of survivors of endometrial cancer.^10^ Much of this research focused upon physical activity, although some aspects of diet were explored. Dietary changes were often motivated by improving health and avoiding bowel side effects and symptoms; social support played an important role in healthy diet motivation, and dietary advice was often sought from both professional and other types of sources. However, endometrial cancer survivors' diets have been noted to differ from other survivors' diets^11,12^ and these changes may not be representative of other cancer types.

Cognitive impairment is a predictor of dietary-related health behaviors,^13^ and is associated with poorer health self-care and dietary management.^14^ Up to 35% of cancer survivors continue to experience cognitive difficulties that persist for years following treatment.^15^ Survivors with cognitive impairment have different self-regulatory styles for managing health conditions through diet and exercise, compared with survivors without cognitive impairment.^16^ Cancer-related cognitive impairment can be identified through subjective (self-report) or objective (neuropsychological assessment) methods, however, they are often poorly correlated: survivors frequently report cognitive difficulties despite an absence of poor performance on cognitive assessments.^17,18^

This could be because self-report measures assess performance over a period of time, as opposed to the time of assessment only, or because they are less sensitive to impairment affecting daily activities. Regardless, self-reported cancer-related cognitive impairment is seen as a valuable and important way of identifying survivors' perceptions of subtle, but meaningful, cognitive changes that impact survivors on a day-to-day basis.^19^

Considering the few studies exploring survivors' motivations of dietary habits with the importance of cognitive impairment on health behaviors, this highlights gaps in understanding dietary motivators and challenges of survivors of other cancer types, and among survivors who experience cognitive impairment. Since (1) diet plays an important role in survivorship outcomes; (2) survivors' diets often change over time; and (3) cognitive function impacts dietary behavior, it is important to investigate the factors contributing to dietary changes and what prevents engaging in healthy eating practices among survivors with cognitive impairment specifically.

This study therefore aims to qualitatively identify the perceived causes or drivers of “prediagnosis to post-treatment” dietary habits, and the barriers preventing survivors engaging in positive dietary practices. To contextualize these perceived drivers and barriers, we also describe the main ways by which participants reported that their eating habits had changed, comparing current with prediagnosis dietary habits.

## Materials and Methods

This article reports on a subset of participants from a previous study, which explored the perceived relationships between diet and cancer-related cognitive impairment among breast (*n* = 13) and colorectal (*n* = 2) cancer survivors.^20^ Gastrointestinal cancers (such as colorectal cancer) and their treatments may directly impact survivors' digestive function and dietary habits substantially differently from survivors of breast cancer, where the digestive system is less commonly affected by both the cancer and its treatments.^21^ Since the current study specifically investigates dietary changes and challenges, and the small number of colorectal cancer survivors did not allow for comparison of themes between groups, we here report findings from the breast cancer survivors only.

### Design

A qualitative framework was chosen to elicit survivors' subjective perceptions and experiences to provide rich data from which to begin understanding the research topic in this specific population.^22^ The intention was to identify self-reported dietary challenges and causes, to identify unique themes that could inform future research. Semistructured interviews were chosen to provide question consistency across participants, while allowing flexibility to explore participant answers in depth. This was particularly relevant among a population experiencing self-described cognitive difficulties, where a variety of prompts and wordings could be used. Data were analyzed with a contextualist approach and using thematic analysis as suggested by Braun and Clarke.^23^ The 21-item “Standards for Reporting Qualitative Research”^24^ was used to inform the study methodology. Ethics approval was obtained from the University of South Australia Human Research Ethics Committee.

### Recruitment

Participants were recruited through community notice boards, online social media, and cancer support groups. Individuals were telephone screened to determine eligibility. Included participants had been diagnosed with adult breast cancer in the past 5 years; were not on or anticipating to undergo primary cancer treatments; and had self-reported cognitive changes after diagnosis. Eligibility relating to cognitive changes required participants to have perceived one or more ways in which their cognition (such as memory, thinking ability, concentration, or decision-making) had worsened since being diagnosed with cancer. Individuals were excluded if they had been diagnosed with any cancer other than breast cancer that required treatment beyond surgical resection/excision, or had experienced significant head trauma.

### Procedures

Eligible participants were sent an information sheet and a consent form. Participants were asked to complete a single interview, and given the option to complete it through telephone or in person at the university campus, according to participant convenience. The study's purpose and procedures were explained and written informed consent obtained for all participants.

Basic demographic and treatment history data were collected. The semistructured format broadly explored the following topics, sequentially: (1) persisting cognitive changes observed since diagnosis currently experienced, and the impact they had; (2) current dietary habit (including how it presently differed from prediagnosis), and the factors contributing to dietary changes or prevented making dietary changes; and (3) the perceived impact diet and cognition had on each other. Participants were asked how their cognitive function was different or had changed since diagnosis (and following treatment cessation) to explore the perceived long-term effects of cancer upon survivors' cognition.

The semistructured question format (pertaining to the Results section) and the findings of this study focused on current dietary habits, changes to diet since diagnosis and treatment cessation, and factors perceived to contributing to those changes. All interviews were audio recorded (SONY recorder ICD-UX200F), conducted by D.G.C. The interviewer engaged in written critical reflections after each interview to monitor and improve research practices. Interviews lasted for ∼45–60 minutes. Participants were given a $20 gift card and the opportunity to review their interview transcript to amend their responses. One participant made minor grammatical changes.

### Data analysis

Demographic data were entered into Microsoft Excel and audio files transcribed into Microsoft Word. NVivo software (Plus v12)^25^ was used to code and analyze transcripts. The researchers consulted each other to identify the themes central to the research questions addressed in this article. Transcript coding and initial theme development were completed by D.G.C. (the interviewer), utilizing a reflexive approach^26^ using data from all interviews. These themes were refined through discussion of sample participant quotes among the research team (D.G.C., A.D.H., and A.M.C.), which were then used to code all data for all participants *de novo* (by D.G.C.). Data from the final two interviews did not require creation of additional themes, demonstrating data saturation.

All coded data relating to themes presented (in the Results section) were reviewed by a second team member (A.M.C.) to ensure appropriate coding to the context and meaning of the theme; any disagreements were resolved through discussion. Themes related to dietary changes that were not previously identified in the analysis of the relationship between diet and cognition (see Ref.^20^) are reported.

## Results

### Participant characteristics

**FIG. 1.**
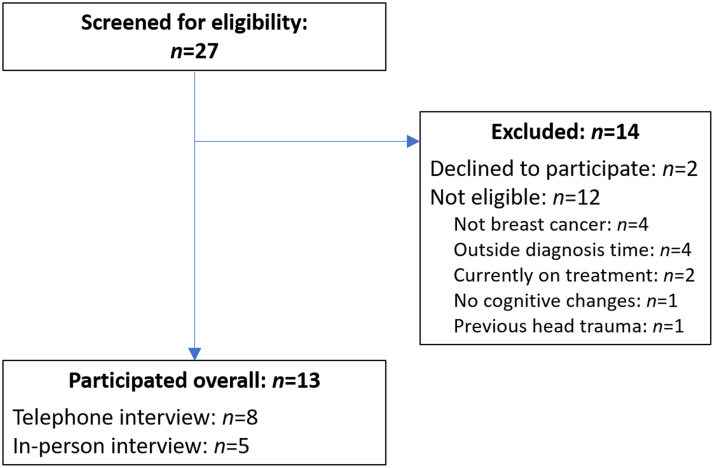
Consort diagram of recruitment and participation.

The number of participants who were screened and completed this study is summarized in Figure 1, with demographic details presented in Table 1.

**Table 1.** Demographic and Treatment Characteristics of Participants

Demographic details	Total sample (*n* = 13)
Mean age in years, (SD) (range)	51.5 (10.4) (27–69)
Sex, *N* (%)	
Female	13 (100)
Ethnicity, *n* (%)	
White	13 (100)
Marital status, *n* (%)	
Single	2 (15)
Married or de facto relationship	11 (85)
Highest educational status, *n* (%)	
Primary school	1 (8)
Secondary school	2 (15)
Graduate/undergraduate degree	7 (54)
Postgraduate degree	3 (23)
Current employment, *n* (%)	
Not employed	1 (8)
Part-time	6 (46)
Full-time	4 (31)
Retired	2 (15)
Treatment history, *n* (%)	
Surgery	12 (92)
Radiotherapy	10 (77)
Chemotherapy	11 (85)
Treatment characteristics Mean time in months since (SD) [range]	
Diagnosis	23.6 (15.3) [7–52]
Surgery	24.0 (15.7) [9–52]
Radiotherapy	19.0 (16.2) [1–45]
Chemotherapy	12.6 (12.7) [3–46]
Completing any primary treatment	14.7 (15.3) [1–45]

Participants described ways in which their diet or eating habits changed compared with prediagnosis. Acute dietary changes that occurred during primary treatment were not included here, as only long-term changes persisting into post-treatment survivorship were relevant to this study's aim. Themes were classified as follows: (1) changes in eating habits since diagnosis; (2) perceived drivers of eating habit changes; and (3) barriers to making intentional dietary changes. As participant responses often involved biopsychosocial complexities and touched upon intersecting issues, themes were not mutually exclusive, and data could be coded to more than one theme.

### Changes in eating habits

Three themes related to how survivors identified that their current eating habits differed from prediagnosis: (1) “I eat when I feel like it” (meal timing shifts); (2) “swap out the meat” (more plant-based eating); and (3) “throwing meals together” (less variety, greater convenience). Examples of participant quotations corresponding to each theme are listed in Table 2, followed by details of each theme. While most participants (*n* = 8) described their diet as somewhat comparable with prediagnosis (largely perceiving their diet to have been relatively good), they identified specific dietary components that had changed, and were therefore coded into relevant themes. Others identified overall improvements (*n* = 2), declines (*n* = 2), and one was equivocal.

**Table 2.** Changes in Eating Habits: A Summary of Themes and Related Quotes

Theme [no. of participants mentioned]	Example participant quote (participant no.)
“I eat when I feel like it” (meal timing shifts) (7/13)	“Yeah, changed the way we eat a bit… [we now have] a bigger meal at lunchtime and go do things and a smaller meal at night…far more likely to have a lighter more protein meal at night…” (P09)
“Swap out the meat” (more plant-based eating) (7/13)	“…more vegetable based now… I sort of like make some vegetable patties, that I might have with salad, instead of a piece of chicken with salad for example.” (P03)
“Throwing meals together” (less variety, greater convenience) (5/13)	“It's reaching for whatever I can do quickly…often running short of things, because I haven't thought [about what to cook] …it's in the too hard basket.” (P01)

#### “I eat when I feel like it” (meal timing and frequency shifts)

Changes to timing of eating were very common (*n* = 7). Specific changes varied: some were viewed positively and empowering, such as having greater flexibility to eat earlier in the day, smaller meals more frequently, or grazing; others were viewed negatively, including late-night snacking and accommodating treatment side effects (*i.e.*, eating earlier to avoid reflux).

#### “Swap out the meat” (more plant-based eating)

Several participants shifted toward utilizing more fruits, vegetables, and legumes, or consuming less red meat in their diets (*n* = 7). This included occasionally substituting meat for plant-based alternatives or fish without specifically adopting a vegetarian diet. These participants were those often working less than before diagnosis, having more time to focus on dietary quality; this contrasted with participants who ate less overall variety [see the “Throwing Meals Together” (Less Variety, More Convenience) section].

#### “Throwing meals together” (less variety, more convenience)

Multiple participants characterizing their diet as more reliant on convenient foods and having less variety compared with before cancer diagnosis (*n* = 5). This included less overall meal variety (*e.g.*, a smaller repertoire of recipes) and reduced variety within meals (*e.g.*, preparing meals with fewer ingredients). Meals were often described as being “thrown together” at the last minute. Lack of time, mental effort, and tiredness were the commonly attributed reasons for these changes. For two participants, this was connected with self-blame or guilt about not being able to prepare “better” meals.

### Perceived drivers of eating habit changes

Five themes related to what survivors perceived had contributed to changes in eating habits: (1) “I wish I could taste something” (persisting treatment-related changes); (2) “teamwork” (help and support from others); (3) “I got used to not eating” (old treatment habits); (4) “looking after myself” (preventative health and self-care); and (5) “not working 9 to 5” (changes to work schedule). These are summarized in Table 3 with a corresponding participant quote.

**Table 3.** Perceived Drivers of Eating Habit Changes: A Summary of Themes and Related Quotes

Theme (no. of participants mentioned)	Example participant quote (participant no.)
“I wish I could taste something” (persisting treatment-related changes) (10/13)	“I have no taste as a result of chemo and so that makes things quite difficult to find something which is appealing. So, I couldn't actually care whether I ate pumpkin soup for dinner ten nights in a row…And that is a huge part of the changes in my life.” (P09)
“Teamwork” (help and support from others) (7/13)	“I struggle with it when I'm by myself at home. If my husband's here, it's a different story because we egg each other into making sure that we eat healthily” (P14)
“I got used to not eating” (old treatment habits) (5/13)	“And I ‘spose from not being very well having the chemo and everything, I've just got used to not eating. ‘Cause then I couldn't keep some foods down and things like that, so…I've sort of changed…[I'm] not that hungry and not that motivated…” (P02)
“Looking after myself” (preventative health and self-care) (5/13)	“…as a cancer survivor, you need to really focus on your wellbeing… how your body you get energy from your food…I like to buy good quality food…fresh things like that…Having cancer almost gives you permission to say, well, to look after yourself.” (P13)
“Not working 9 to 5” (changes to work schedule) (5/13)	“…most of what I cook is from scratch, and has mostly always been the case, but it's more the case now, and again because I have the time with not, you know, having to fit everything around work.” (P03)

#### “I wish I could taste something” (persisting treatment-related changes)

Almost all participants attributed changes in their diet to treatment-related symptoms or side effects (*n* = 10), most commonly changes in taste, fatigue, and weight gain. Diminished taste was often reported to reduce enjoyment of food, which contributed to a “dietary apathy” (*e.g.*, “food's not as important as it used to be”—P2).

#### “Teamwork” (help and support from others)

More than half of participants described how other people assisted making positive changes or maintaining positive dietary practices (*n* = 7). This was primarily participants' partners taking on additional roles in meal planning and preparation, though also included receiving dietary advice from others. Even small contributions, such as receiving recipes or sharing meals with others, were perceived as meaningful and positively impactful. This contrasted with three additional participants' experiences who described being completely responsible for meal preparation and having less external support, which was a significant challenge in their dietary habits. In contrast, another participant identified that while receiving assistance from her husband was helpful, it could negatively impact her diet (“I think I was more in control of what we were eating…we're mainly just having red meat and 3 veg… and they're more low-quality vegetables rather than ones that…are more beneficial”—P12).

#### “I got used to not eating” (old treatment habits)

For some, behavioral patterns and habits that they initiated during chemotherapy persisted beyond treatment cessation (*n* = 5; mean time since cessation for these participants was 16 months). This included continuing to avoid food that had caused physical discomfort during treatment, even though they no longer caused discomfort, or continuing to avoid foods they were advised not to eat during treatment. One participant described no longer being interested in eating, having habituated to not feeling hungry during chemotherapy.

#### “Looking after myself” (preventative health and self-care)

Six participants described intentionally making dietary changes to be healthier. This included prevention of cancer recurrence and mitigating potential long-term side effects of medication such as osteoporosis and cardiovascular disease. However, it also included psychological self-care and the importance of prioritizing one's well-being as a survivor.

#### “Not working 9 to 5” (changes to work schedule)

Altered work commitments such as working less or no longer working (largely resulting from cancer-related cognitive difficulties and cancer-related fatigue) were perceived to impact survivors' diets (*n* = 5) in mostly beneficial ways. This allowed participants additional time to focus on and prepare food, or gave greater flexibility with meal timings that better suited their biopsychosocial needs (“…because I'm not working. So I do actually concentrate a lot of my time on my diet”—P10).

### Barriers to making intentional dietary changes

Barriers referred to factors that prevented or made it difficult for participants to initiate dietary behaviors, largely with a “positive” or “healthy” context. Three additional themes emerged relating to barriers: (1) “whatever's quick and easy” (too much time and effort); (2) “life's too short, eat the donut” (food cravings and enjoyment); and (3) “out of ideas” (lacking dietary resources). These are summarized in Table 4, with example participant quotes.

**Table 4.** Perceived Barriers to Intentional Dietary Changes: A Summary of Themes and Related Quotes

Theme [no. of participants mentioned]	Example participant quote (participant no.)
“Whatever's quick and easy” (too much time and effort) (8/13)	“…just the last few days I've got my [cook]books out again…I couldn't…found it difficult to plan things at that level. Yes, I could walk to the shop and go and grab a few things, but if I had to sit down and really plan it, it was too much…” (P13)
“Life's too short, eat the donut” (food cravings and enjoyment) (8/13)	“I'm a food scientist…so I knew the impact of foods and what I needed to eat…Post-[radiation], I'd found an absurd drive for sweet foods… it was manic… looking for any form of sugar that I could find… if it was there, I'd eat it…” (P12)
“Out of ideas” (lacking dietary resources) (4/13)	“I would love if they had some sort of place you could go to that said… “we're going to do a cooking class today and this is going to focus on good food, fresh food, easy to get on the table, and it's not going to cost you $500 to do the session.” That'd be great! Walk away with 4 or 5 different recipes that didn't have weird ingredients.” (P1)

#### “Whatever's quick and easy” (too much time and effort)

Lack of time and requiring too much effort prevented most participants from making intentional dietary changes and improvements (*n* = 8). This largely involved the competing demands of broader life responsibilities and commitments, which had emerged or had become more taxing following cancer diagnosis, particularly from professional and work commitments. For example, taking longer to do things and its subsequent time pressures, being more tired at the end of a “normal day,” and being unable to plan or prepare meals as they could prediagnosis. This contrasted with survivors who reported working less [see theme “Not Working 9 to 5” (Changes to Work Schedule) section] had perceived beneficial effects on diet.

#### “Life's too short, eat the donut” (food cravings and enjoyment)

Desire for certain foods (largely described as “unhealthy”) was common (*n* = 8). For some survivors, taste changes led to seeking out specific foods such as chocolate, sugar, salt, and spicy foods. Some participants described difficulties with sugar cravings postdiagnosis; one participant experienced improvements after being prescribed diabetic medication (although not having diabetes). Two participants mentioned the importance of enjoying discretionary foods postdiagnosis (*e.g.*, wine and donuts) to “live life,” while one participant noted healthier foods were simply no longer appealing.

#### “Out of ideas” (lacking dietary ideas and resources)

Generating ideas of what to cook was problematic for four participants. In addition to not having adequate time or energy [theme “Whatever's Quick and Easy” (Too Much Time and Effort) section ], it also involved the challenge of finding recipes and methods of cooking with an appropriate level of ease to meet their new physical and mental abilities. Being met with external resistance also played a role, such as feeling unable to provide or prepare healthier foods while navigating and accommodating family members' taste preferences.

## Discussion

This study explored the prediagnosis to post-treatment changes in eating habits of breast cancer survivors with self-reported cancer-related cognitive impairment; it examined survivors' perceptions of the drivers of those changes, and the experienced barriers that prevented making intentional post-treatment dietary changes.

In this group, survivors identified three primary changes to eating habits. The most commonly reported change was a shift toward plant-based eating. This greater inclusion of fruits and vegetables, and reduction in red meat, follows dietary recommendations for survivors by the World Cancer Research Fund.^3^ This also aligns with previous quantitative studies reporting increased postdiagnosis fruit and vegetable intake.^27,28^

However, contrasting this and contrary to these recommendations were a reduced dietary variety and reliance on more convenient foods for others. Increased use of processed foods can lead to a greater intake of saturated fat, sugar, and sodium,^29^ generally recommended to be limited in a healthy diet.^30^ In addition, reduced dietary diversity is associated with a higher all-cause mortality.^31^ The participants who had reduced work ability (due to cognitive difficulties and fatigue) often reported having more time to focus on dietary improvements (*e.g.*, plant-based eating), compared with those who often found themselves “taking short cuts” using reduced variety and more convenience foods.

Employment changes were also described as impacting meal timing shifts among many survivors in this study; this was the other most experienced dietary change. Decreased or ceased work participation is more common in cancer survivors compared with noncancer populations,^32^ which can have both positive^33^ and negative^34^ dietary consequences. In the current study, timing changes were largely regarded as positive, giving more flexibility and freedom of when survivors could choose to eat. However, changes in meal timing and distribution can have negative consequences on mood, sleepiness, and cognitive performance.^35–37^ While the area of has been explored in relation to cancer risk,^38^ it may be worth expanding upon in relation to quality of life and cognitive function in survivors of cancer. The continually changing aspects of work and daily routines across survivorship stages are therefore an important component in understanding and supporting survivors' dietary habits and needs, differentially to the general population.

Survivors in this study spoke about experiencing several post-treatment challenges influencing dietary changes: persisting treatment-related changes in taste and fatigue were the most commonly attributed causes. In previous research, half of the survivors with cancer-related fatigue reported fatigue negatively affecting meal preparation, with five percent requiring professional assistance.^39^ Fatigue is commonly experienced in 50–90% of all cancer survivors,^40^ and is a robust factor influencing self-reported cognitive impairment in survivors.^17^ Little research has explored dietary changes and challenges of survivors with cancer-related fatigue specifically, but due to its prevalence among survivors, should be explored. While taste changes are common in cancer survivors,^41^ they are not known to be linked with cognitive impairment^42^ and likely to be a broader survivorship challenge.

In this sample though, altered taste was linked to food enjoyment and contributed to craving “unhealthy” foods such as chocolate, salt, sugar, and “junk food.” Consideration of taste changes may therefore be helpful when assisting cancer survivors to achieve or maintain healthy eating habits.

An interesting behavioral theme emerged with some survivors in this study reporting that dietary changes made during treatment persisted beyond treatment cessation. In many cases, these were changes to address symptoms that no longer caused them discomfort but had become a learned behavior and integrated in routines. Even small dietary changes resulting from health conditions such as reduced hunger, taste changes, or mild dementia onset can contribute to significant shifts in macro- or micronutrient intakes that can have subsequent health effects.^43^ Survivors in this sample may have benefited from receiving nutritional review and guidance at some point following primary treatment completion, to create and support healthy and appropriate dietary plans.

Future research should explore whether a post-treatment review of healthy dietary goals has usefulness and is important, particularly when survivors report cognitive changes or difficulties. This is especially important in light of recent research identifying that cancer survivors with cognitive impairment are more likely to rely on external cues and sources of motivation for self-managed health-related lifestyle behaviors, than survivors without cognitive impairment.^16^ Participants in the current study varied regarding when they would have wanted to receive nutritional information (*e.g.*, during vs. after treatment), representing survivors' heterogenous needs and experiences of cancer, congruent with previous research.^5,10^

This study found that lack of time and energy (compared with prediagnosis) was a common barrier to making intentional dietary changes. Meals that were not “quick and easy” were less likely to be prepared postdiagnosis among survivors in this study, due to competing demands and fatigue. However, research identifies that cooking and meal planning can form important parts of individuals' self-identity and their role within their social units; cancer survivors can find it distressing when they can no longer engage in culinary routines as they did prediagnosis.^44^ Nevertheless, social and family support is often critical in enabling cancer survivors to make and maintain positive dietary changes.^10,45^

Related to this, engaging in positive dietary behaviors as a form of self-care was a reported driver of eating habits, in conjunction with preventative health. While previous qualitative work reveals preventative health to be a common motivation for dietary change following diagnosis,^46^ the additional emphasis of self-care in this study highlights the psychological importance of food in survivorship, beyond biological nutrition. This may be related to this sample's experiences of cognitive impairment, and the ensuing psychosocial limitations and challenges that require additional navigation and self-care. The need for social supports or interventions among survivors experiencing diminished cooking and meal preparation capabilities and for whom cooking is important should be further explored. Interventions with cooking classes can improve cancer survivors' psychological well-being^47,48^ and can bolster well-being, however, effectiveness of these interventions has not yet been assessed in individuals with cognitive impairment.

This relates to one of the barriers of dietary changes reported by participants: the need for recipes and meal plans that were quick and simple, family-friendly, and could be prepared in advance if needed, to accommodate being time-poor, and physically and cognitively exhausted. While this theme related to fewer participants overall, multiple other participants identified that being given recipes or cooking ideas from others had been a positive support. Multiple studies have explored the benefits of cooking classes on survivors'^49–51^ however, few have investigated dietary resources such as recipe books and meal plans. Given that one of the most common challenges in this study was lack of time, we suggest investigation is needed into the feasibility and effectiveness of these resources, particularly for survivors who experience challenges relating to cognition and fatigue.

Limitations of this study must be considered. Participants were all Caucasian breast cancer survivors, who were by and large highly educated, limiting generalizability. Importantly, this study dealt with perceptions; cancer survivors' perceptions of diet quality may not reflect true dietary intake. The limitations of self-report dietary measures have been demonstrated in previous research with cancer survivors^52^; in addition, these authors identified the need for dietary counseling and education to improve dietary knowledge to foster more accurate dietary self-assessment, and in turn dietary quality. Without comparison groups, we cannot identify whether the findings in this study are specific to survivors with cognitive impairment, apply to survivors in general, or are generalizable to noncancer and healthy populations. As participants self-identified having cognitive difficulties, issues regarding recalling relevant information during a single interview session may have prevented important information being shared.

While survivors were given the opportunity to review and amend their interview transcript and cognitive difficulties were usually reported to be mild, future research could include interviews with partners or family members to gain additional perspectives. Due to the small sample size and exploratory nature of this qualitative study, more research is needed to explore the degree and ways by which the presented themes are prevalent and relevant to different survivorship populations and to expand on these findings.

## Conclusions

Cancer survivors face unique circumstances and limitations, such as physical and mental fatigue, disruptions to routines and schedules, and navigating interpersonal responsibilities that uniquely influence their dietary behaviors. It is important to find ways to support survivors to maintain and engage in healthy dietary practices, particularly ways that accommodate survivorship difficulties that may accompany perceived changes to cognitive abilities following cancer diagnosis, which are meaningful and achievable for survivors themselves. This may include access to dietary resources and nutritional review with qualified health professionals in post-treatment survivorship care. Future research could expand on this study by examining the impact of these dietary changes on survivors' health and quality of life and exploring strategies to overcome dietary challenges associated with cancer survivorship.

## Abbreviation Used

SD: standard deviation
